# GFR is a Key Determinant of Red Blood Cell Survival in Anemia Associated With Progressive CKD

**DOI:** 10.1016/j.ekir.2024.12.023

**Published:** 2024-12-21

**Authors:** Rosi Bissinger, Lina Schaefer, Bernhard N. Bohnert, Anja Schork, Sebastian Hoerber, Andreas Peter, Syed M. Qadri, Andreas L. Birkenfeld, Nils Heyne, Tamam Bakchoul, Thomas Wieder, Ferruh Artunc

**Affiliations:** 1 Department of Internal Medicine, Division of Diabetology, Endocrinology and Nephrology, University Hospital Tübingen, Tübingen, Germany; 2 Institute of Diabetes Research and Metabolic Diseases of the Helmholtz Center Munich at the University of Tübingen, Tübingen, Germany; 3 German Center for Diabetes Research at the University of Tübingen, Tübingen, Germany; 4 Department for Diagnostic Laboratory Medicine, Institute for Clinical Chemistry and Pathobiochemistry, University Hospital of Tübingen, Tübingen, Germany; 5 Faculty of Health Sciences, Ontario Tech University, Oshawa, Ontario, Canada; 6 Innovation & Portfolio Management, Canadian Blood Services, Ottawa, Ontario, Canada; 7 Institute for Clinical and Experimental Transfusion Medicine (IKET), University Hospital of Tübingen, Tübingen, Germany; 8 Centre for Clinical Transfusion Medicine, University Hospital of Tübingen, Tübingen, Germany; 9 Department of Vegetative and Clinical Physiology, University of Tübingen, Tübingen, Germany

**Keywords:** anemia, chronic kidney disease, eGFR, eryptosis, phosphatidylserine, red blood cells

## Abstract

**Introduction:**

Anemia is a common and clinically significant complication observed in patients with chronic kidney disease (CKD), resulting from complex interactions between renal dysfunction, erythropoietin (EPO) deficiency, and altered iron metabolism. In murine CKD models, red blood cell (RBC) death or eryptosis, characterized by exposure of phosphatidylserine (PS) on the outer membrane of RBCs, was observed to drive anemia. However, there is limited research that has investigated this phenomenon in patients with non–dialysis-dependent CKD (NDD-CKD).

**Methods:**

In this cross-sectional cohort study, we describe the relationship between RBC death and anemia in all stages of NDD-CKD (*n* = 122). Blood samples from 133 healthy blood donors were additionally analyzed as controls.

**Results:**

Patients with CKD had a significantly lower hemoglobin (Hb) concentration (12.4 [interquartile range: 11.1–13.7] g/dl) when compared with the healthy group (13.8 [13.0–14.8] g/dl, *P* < 0.001). Hb concentrations exhibited a significant positive correlation with the estimated glomerular filtration rate (eGFR) across the entire cohort (*r* = 0.5, *P* < 0.001). RBC death rates, quantified by the binding of freshly isolated RBCs to the ligand annexin V using flow cytometry (FACS), were significantly increased by approximately 1.4-fold in patients with CKD compared with the RBC death rates in healthy blood donors. RBC death correlated with the glomerular filtration rate (GFR) stage but not with the albuminuria stage of CKD, the degree of anemia, and serum iron concentration. Using multiple linear regression, eGFR was identified as the sole independent predictor of RBC death with an inverse relationship.

**Conclusion:**

RBC death is stimulated in progressive NDD-CKD, possibly contributing to the development of renal anemia.

## Graphical abstract

**Figure:**
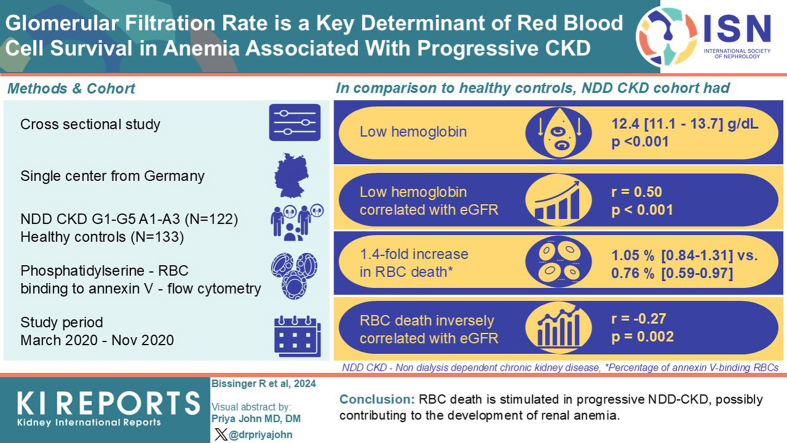

With a global prevalence of about 10% to 15% of the population,^1^^,^^2^ CKD is a complex and long-lasting health condition. Among the individuals suffering from CKD, a notable proportion experience anemia, defined by a decrease in the Hb concentration below 12.0 g/dl in females and 13.0 g/dl in males.^3^ Clinically, the effects of anemia on individuals with CKD are debilitating because it noticeably diminishes both their physical capabilities and overall quality of life.^4^, ^5^, ^6^ Anemia is linked to a worse overall prognosis^7^ and is associated with a faster progression of CKD.^6^^,^^8^^,^^9^ Moreover, it contributes to elevated morbidity and mortality rates,^9^, ^10^, ^11^ increased cardiovascular risk,^12^ amplified cognitive disorders,^13^ more frequent hospitalizations,^8^^,^^11^ and greater financial costs.^14^

Renal anemia presents itself as hypoproliferative, normochromic, and normocytic.^3^ Its etiology is multifactorial^12^ and relates to a progressive decline in endogenous EPO synthesis,^15^ iron deficiency,^16^ a compromised bone marrow response because of exposure to uremic toxins and inflammation, and deficiencies in vitamin B_12_ or folic acid. The pathophysiology of renal anemia is further confounded by hypothyroidism, hemolysis, hyperparathyroidism, malignancies, and instances of malnutrition.^3^^,^^6^^,^^15^^,^^17^ In recent years, there has been a growing body of evidence showing that RBC death is a potentially important contributing factor for the development of renal anemia.^18^ RBC death or eryptosis hereby alludes to the process of accelerated decay and subsequent elimination from circulation by macrophages.^18^, ^19^, ^20^ This process is facilitated by exposure of PS on the outer layer of the plasma membrane, constituting an “eat me” signal.^19^ These PS-exposing RBCs can be readily quantified using FACS after binding to its ligand annexin V.^21^

In CKD, RBC lifespan is typically reduced from approximately 122 days in stage G1 to approximately 60 days in stage G5,^22^^,^^23^ which may be accounted for by expedited RBC death and clearance. Indeed, anemia in 2 mouse models of proteinuric CKD was driven by a markedly reduced RBC lifespan because of stimulation of RBC death.^24^ High-throughput metabolomic analyses of RBCs demonstrated disturbances in redox recycling pathways and the Lands’ cycle, which is a membrane lipid remodeling process involving phospholipids.^24^

In patients undergoing hemodialysis or peritoneal dialysis, our group has demonstrated stimulation of RBC death;^25^^,^^26^ however data showing increased RBC death in patients with NDD-CKD is limited. Although a few studies have been published,^27^, ^28^, ^29^ these studies are limited by their relatively small sample size and the lack of further analyses for contributing factors that might be correlated with enhanced RBC death. Therefore, we conducted a cross-sectional study to investigate RBC death and its relation to anemia in patients with NDD-CKD and other factors across all stages.

## Methods

### Study Cohort

This study included a cohort of stable outpatients with CKD, who sequentially attended routine follow-up appointments at the University Hospital of Tübingen, Germany from March 2020 to November 2020. From a total of 153 patients, 31 were excluded because of the following reasons: (i) unconfirmed CKD diagnosis (*n* = 27), (ii) dialysis at the time of blood collection (*n* = 1), (iii) acute-on-chronic renal failure (*n* = 2), and (iv) i.v. iron therapy shortly before the measurement (*n* = 1). Ultimately, the study included a total of 122 patients (Table 1). From each patient, 10 ml lithium-heparinized blood and spot urine samples were collected. In addition, on the same investigation day, blood samples from 133 healthy age-matched subjects from the Center for Clinical Transfusion Medicine in Tübingen were procured. eGFR assessments were performed in both the patient and healthy control groups at the central laboratory of the University Hospital. The ethical clearance for this study was granted by the local ethics committee of the University Hospital Tübingen (556/2018 BO2), and the study was executed in adherence to the principles outlined in the Declaration of Helsinki. Both patients and healthy volunteers provided their informed consent.

**Table 1:** Demographic and clinical characteristics of the study participants

Parameter	Patients with CKD (*n* = 122)	Healthy controls (*n* = 133)	*P*-value
Sex	62 males (51%)	62 males (47%)	0.5855
	60 females (49%)	71 females (53%)	
Age, yrs, median (IQR)	58 (43–71)	55 (33–61)	0.0013
BMI, kg/m^2^, median (IQR)	26.4 (24.2–31.1)	n.d.	
Causes of CKD	Glomerular disease (*n* = 48), Diabetic nephropathy (*n* = 9), Hypertensive nephropathy (*n* = 16), Polycystic kidney disease (*n* = 13), Other causes *(n* = 30), Unknown (*n* = 6)	n.a.	
Diabetes mellitus, n (%)	36 (30%)	n.a.	
Arterial hypertension, n (%)	84 (69%)	5 (4%)	
Cardiovascular disease, n (%)	49 (40%)	n.a.	
Medication, n (%):			
RAAS blockade	89 (73%)	4 (3%)	
diuretics	73 (60%)	1 (1%)	
iron	16 (13%)		
ESA	9 (7%)		

BMI, body mass index; CKD, chronic kidney disease; ESA, erythropoiesis stimulating agent; IQR, interquartile range; n.a. not applicable; n.d., no data; RAAS, renin-angiotensin-aldosterone system.

### Isolation of RBCs

To isolate RBCs, 500 μl of lithium-heparinized blood from both patients with CKD and healthy blood donors serving as control samples were transferred into 1.5 ml of Ringer’s solution. This mixture was then gently layered over 2 ml of Pancoll human separating solution, density 1.077 g/ml (PAN Biotech, Aidenbach, Germany). The subsequent step involved centrifugation at 120 relative centrifugal force (rcf) at 20 °C for 20 minutes, followed by the removal of the supernatant. For washing, 2 ml of Ringer’s solution was added, followed by centrifugation at 120 rcf at 20 °C for 10 minutes. After removal of the supernatant, the purified RBCs were used for flow cytometric measurements using a BD FACSCalibur (BD Biosciences, Heidelberg, Germany).

### Determination of PS-Exposure of RBCs and Intracellular Ca^2+^ Concentration

To determine the abundance of PS on the erythrocyte surface, the property of its binding to its ligand annexin V–fluorescein-isothiocyanate was used. To this end, 2 μl of freshly collected blood was mixed with 500 μl of Ringer’s solution containing 5 mM CaCl_2_ and stained with annexin V–fluorescein-isothiocyanate (1:200 dilution; ImmunoTools, Friesoythe, Germany) at 37 °C for 15 minutes under light protection. Annexin V presence on the erythrocyte surface was then analyzed using the BD FACSCalibur. Excitation and emission were set at 488 nm and 530 nm, respectively. A threshold marker (M1) was established to distinguish annexin V-binding cells from control cells. This threshold was applied uniformly to both healthy erythrocytes and those from patients with CKD. A typical histogram is shown in Supplementary Figure S1.

One of the main hallmarks of RBC death is the increase of the intracellular Ca^2+^ concentration in erythrocytes.^19^ To measure the intracellular Ca^2+^ concentration, 2 μl of freshly collected blood was added to 500 μl of Ringer's solution with 5 mM CaCl_2_. The mixture was then stained with Fluo-4, AM ester (5 μM; Biotium, Hayward, CA) and incubated at 37 °C for 30 minutes. The Ca^2+^-dependent fluorescence intensity was assessed using FL-1, with an excitation wavelength of 488 nm and an emission wavelength of 530 nm on the BD FACSCalibur. Following this, the geometric mean of the Ca^2+^-dependent fluorescence was calculated.

### Determination of Reticulocyte Numbers

Reticulocyte count was measured to assess the rate of reticulocyte production in the blood, which is an indicator of bone marrow activity and erythropoietic response to various conditions. For the determination of reticulocyte count, 2 μl of lithium-heparin whole blood was added to 500 μl of BD Retic-Count reagent (thiazole orange; BD Biosciences, Heidelberg, Germany). Samples were stained for 30 minutes at room temperature in the dark. Subsequently, thiazole orange fluorescence intensity (in FL-1) of the blood cells was measured using FACS. The number of Retic-Count positive reticulocytes was expressed as a percentage of the total gated erythrocyte population.

### Quantification of Oxidative Stress

Because oxidative stress is an important mediator of RBC death,^19^ oxidative stress was assessed using 2′,7′-dichlorodihydrofluorescein diacetate; 4μl of erythrocytes were mixed with 1 ml of Ringer’s solution. From the resulting cell suspension, 150 μl was centrifuged (1600 rpm for 3 minutes at RT). Cells were then stained with 2′,7′-dichlorodihydrofluorescein diacetate (10 μM; Sigma, Schnelldorf, Germany) in Ringer’s solution at 37 °C for 30 minutes followed by 3 washes in 150 μl of Ringer’s solution each. The stained erythrocytes were resuspended in 200 μl of Ringer’s solution, and reactive oxygen species (ROS)-dependent fluorescence intensity was measured in FL-1 at an excitation wavelength of 488 nm and an emission wavelength of 530 nm using the BD FACSCalibur. Subsequently, the geometric mean of the ROS-dependent fluorescence was calculated.

### Determination of Ceramide Abundance

Ceramide is a mediator of calcium-independent RBC death. The enzyme sphingomyelinase cleaves ceramide from sphingomyelin, enhancing the sensitivity of the scramblase enzyme to calcium effects.^30^ To quantify the abundance of ceramide on the RBC surface, a monoclonal antibody-based assay was deployed essentially as described earlier by Lang *et al*.^30^ Likewise, 4 μl of purified RBCs were diluted in 1 ml of Ringer’s solution. A 100 μl aliquot underwent centrifugation for 3 minutes at room temperature at 570 rcf. Following this, the cells were stained for 1 hour with an anti-ceramide antibody (1:10 dilution, clone MID 15B4; Alexis, Grünberg, Germany) in phosphate-buffered saline containing 0.1% bovine serum albumin at 37 °C. As a secondary antibody, a polyclonal fluorescein-isothiocyanate–conjugated goat anti-mouse IgG- and IgM-specific antibody (1:50 dilution; BD Pharmingen, Hamburg, Germany) was utilized for staining for 30 minutes at 37 °C.

### Measurements Involving Plasma

To test whether the plasma of patients with CKD contains substances that might trigger eryptosis, plasma was obtained by centrifugation (120 rcf, 5 minutes). Subsequently, erythrocytes were obtained by transferring 500 μl of lithium-heparinized blood from both patients with CKD and healthy blood donors into 1.5 ml of Ringer’s solution. This mixture was then gently layered over 2 ml of Pancoll human separating solution, density 1.077 g/ml. The subsequent step involved centrifugation at 120 rcf at 20 °C for 20 minutes, followed by the removal of the supernatant. 2 μl of purified erythrocytes (0 blood group) from healthy young individuals were incubated *in vitro* with 500 μl plasma from patients or healthy volunteers for 24 hours. After 24 hours, the plasma containing the erythrocytes was mixed and 150 μl of the mix were transferred on a 96-well plate. Finally, PS-exposure was determined as described above.

### Laboratory Assays

Laboratory parameters such as hemogram, plasma creatinine and cystatin C concentration, parameters of iron status, and EPO were measured using a Sysmex KX-21N, ADVIA 1800 chemistry system (Siemens Healthineers, Forchheim, Germany), and IMMULITE 2000 immunoassay system (Siemens Heathineers, Forchheim, Germany). In patients with CKD, urine protein and albumin were determined by a turbidimetric benzethonium chloride assay (Roche Diagnostics, Mannheim, Germany) and nephelometric method via a BN ProSpec System (Siemens, Forchheim, Germany), respectively. eGFR was calculated as per the combined CKD Epidemiology Collaboration creatinine-cystatin C formula of 2012.^31^

### Statistical Analysis

Data were presented as either arithmetic means ± SD or as medians accompanied by the first and third quartile, respectively, with “n” indicating the count of patients or healthy volunteers, as applicable. The normality of data distribution was assessed using the Shapiro-Wilk test. A range of statistical tests including *t-*test, Mann-Whitney U-test, analysis of variance, Tukey post hoc test, Kruskal-Wallis test, Bonferroni correction, Wilcoxon test, Pearson correlation test, and multiple regression analysis with a stepwise model for selection of parameters entering a final model, were executed as indicated. A *P*-value < 0.05 was considered statistically significant. Statistical analyses were carried out using R Version 4.0.2 (2020-06-22) and RStudio Version 1.3.1093 (RStudio Team, 2020). Multiple regression analysis was carried out using IBM SPSS Statistics Version 29.0 (IBM Corp, 2022) and MedCalc Version 20.215 (MedCalc Software Ltd, 2023a). In addition, Microsoft Excel Version 2302 (Microsoft Corporation, 2023a) was employed.

## Results

### Study Cohort

The study participants included 122 patients with NDD-CKD and 133 age-and sex-matched (male/female) healthy blood donors serving as controls. The sex distribution was similar in both the groups; however, patients with CKD were slightly older (58 vs. 55 years, *P* = 0.0013, Table 1). The causes and stages of CKD are outlined in Table 1. Comorbidities such as diabetes mellitus, arterial hypertension, and cardiovascular disease were highly prevalent in the patient group (30%, 69%, and 40%, respectively). Sixteen patients (13%) received oral or i.v. iron therapy and 9 (7%) received EPO supplementation.

The median eGFR of the patients with CKD was 33 (interquartile range: 22–48) ml/min per 1.73 m^2^ as compared with 91 ml/min per 1.73 m^2^ (81–105) in the healthy group (*P* < 0.001, Table 2). The distribution of patients with CKD across different stages was as follows: (i) G1: *n* = 8 (7%), (ii) G2: *n*= 12 (10%), (iii) G3a: *n* = 16 (13%), (iv) G3b: *n* = 34 (28%); (v) G4: *n* = 39 (32%), and (vi) G5: *n* = 13 (11%) out of a total of *n* = 122 patients. Median albuminuria was 191 (52–1388) mg/g creatinine, and accordingly 15 (13%) patients with CKD were classified as stage A1, 50 (43%) as stage A2, and 52 (44%) as stage A3. Among the latter, 21 patients with CKD (40% of stage A3 or 17% of all patients) fell within the nephrotic range with proteinuria > 2200 mg/g creatinine. There were no urine samples for 5 patients.

**Table 2:** Laboratory parameters of the study participants

Parameter	Patients with CKD (*n* = 122)	Healthy controls (*n* = 133)	*P*-value
Plasma creatinine, mg/dl (IQR)	1.6 (1.2–2.2)	0.7 (0.7–0.9)	< 0.001
Plasma cystatin C, mg/l (IQR)	2.05 (1.5–2.8)	1.0 (0.8–1.0)	< 0.001
eGFRcr-cys, ml/min per 1.73 m^2^ (IQR)	33 (22–48)	91 (81–105)	< 0.001
Hemoglobin, g/dl (IQR)	12.4 (11.1–13.7)	13.8 (13.0–14.8)	< 0.001
Hematocrit, %	36.3 ± 5.9 (*n* = 114)	42.2 ± 4.9	< 0.001
Erythrocytes, 10^6^/μl	4.17 ± 0.73 (*n* = 114)	4.75 ± 0.6	< 0.001
MCV, fL	87 ± 6 (*n* = 114)	89 ± 4	0.0235
MCH, pg	30 (29–31) (*n* = 114)	29 (28–31) (n = 61)	0.1173
Plasma CRP, mg/dl (IQR)	0.21 (0.04–0.66)	0.06 (0.02–0.15)	< 0.001
Plasma iron, μg/dl (IQR)	72 (56–93)	94 (62–126)	< 0.001
Plasma ferritin, μg/dl (IQR)	7.3 (3.2–15)	2.3 (1.6–4.2)	< 0.001
Plasma transferrin, mg/dl (IQR)	229 (198–257)	275 (259–303)	< 0.001
TSAT, % (IQR)	23 (16–30)	24 (16–33)	0.5278
Plasma EPO, mU/ml (IQR)	10 (7–15)	10 (8–13)	0.8501
Plasma total protein, g/dl	6.8 ± 0.5 (*n* = 85)	n.d.	
Urinary protein, mg/g creatinine (IQR)	313 (140–1275) (*n* = 117)	n.d.	
Urinary albumin, mg/g creatinine (IQR)	191 (52–1388) (*n* = 117)	n.d.	

CKD, chronic kidney disease; CRP, C-reactive protein; eGFR, estimated glomerular filtration rate; EPO, erythropoietin; IQR, interquartile range; MCH, mean corpuscular hemoglobin; MCV, mean corpuscular volume; n.d., no data; TSAT transferrin saturation.

Values are arithmetic means ± SD or medians with interquartile range.

### Anemia in the Cohort

**Figure 1:**
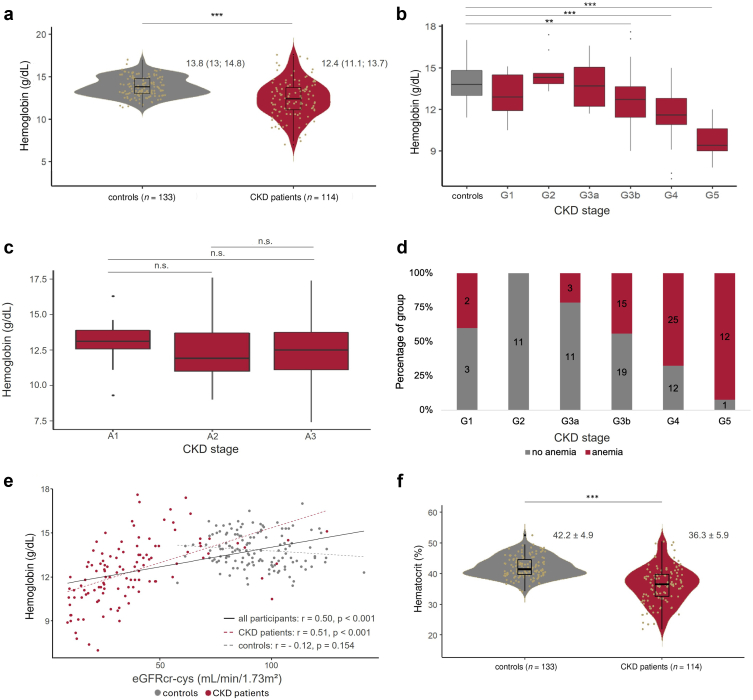
Progressive CKD is associated with anemia. (a) Hemoglobin concentration in the control and CKD group. (b and c) Hemoglobin concentration according to CKD stages G1 to G5 as well as albuminuria stages A1 to A3. (d) Number of anemic versus nonanemic patients in the respective CKD stages. (e) Correlation between hemoglobin concentration and eGFR in all participants, including both the control and CKD groups, as well as within the control and CKD groups separately. (f) Hematocrit values in the control and CKD group. CKD, chronic kidney disease; eGFR, estimated glomerular filtration rate.

As shown in Figure 1a, patients with CKD had a significantly lower Hb concentration when compared with the healthy group. This difference was also evident when comparing the median Hb values of male (13.1 [11.2–14.5] g/dl) and female (11.9 [10.7–12.9] g/dl) patients with CKD with the respective medians of healthy individuals of the same sex (14.8 [14–15.3] g/dl and 13.1 [12.7–13.6] g/dl, respectively, both *P* < 0.001). Reduced Hb concentration correlated with the GFR stage of CKD (Figure 1b), whereas no significant variability was noted across different albuminuria stages of CKD (Figure 1c). As shown in Figure 1d, the proportion of patients with anemia was higher in higher GFR stages. Hb concentrations exhibited a significant positive correlation with eGFR across the entire cohort (*r* = 0.50, *P* < 0.001, Figure 1e), and this correlation was dominated by the correlation within the subgroup of patients with CKD (*r* = 0.51, *P* < 0.001). In the control group, there was no correlation between eGFR and Hb (*r* = −0.12, *P* = 0.154). As for Hb concentrations, the hematocrit among patients with CKD was significantly lower in comparison with the healthy group (Figure 1f). Hematocrit was similarly influenced by GFR stages (37.1% ± 5.2% in stage G1, 41.5% ± 3.5% in stage G2, 39.9% ± 4.8% in stage G3a, 37.7% ± 5.6% in stage G3b, 34.5% ± 5% in stage G4, and 29.2% ± 3.8% in stage G5). The RBCs of the patients with CKD exhibited a significantly lower volume, as indicated by mean corpuscular volume, in comparison with the healthy group (Table 2). However, the mean corpuscular Hb value was similar in both groups.

### Iron Status

The plasma ferritin concentration was significantly higher in patients with CKD as compared with healthy individuals whereas plasma iron and transferrin concentrations were significantly lower (Table 2). Transferrin saturation, however, did not exhibit a significant difference. Both plasma iron and transferrin levels decreased with higher GFR stages, presumably because of increased inflammation suggested by higher C-reactive protein concentration in patients with CKD (Table 2). Among the patients with CKD, 40 (33%) exhibited iron deficiency, as characterized by transferrin saturation ≤ 20% and ferritin ≤ 10 μg/dl^3^ (Supplementary Figure 2A), whereas 24 patients manifested iron deficiency anemia (21%, out of *n* = 114) (Supplementary Figure 2B). These proportions tended to increase with higher GFR stages (Supplementary Figure 2A and B). Among the control individuals, 48 individuals (36%, out of *n* = 133) were identified with iron deficiency, of which only 3 individuals had iron deficiency anemia (2%).

### Markers of Erythropoiesis

**Figure 2:**
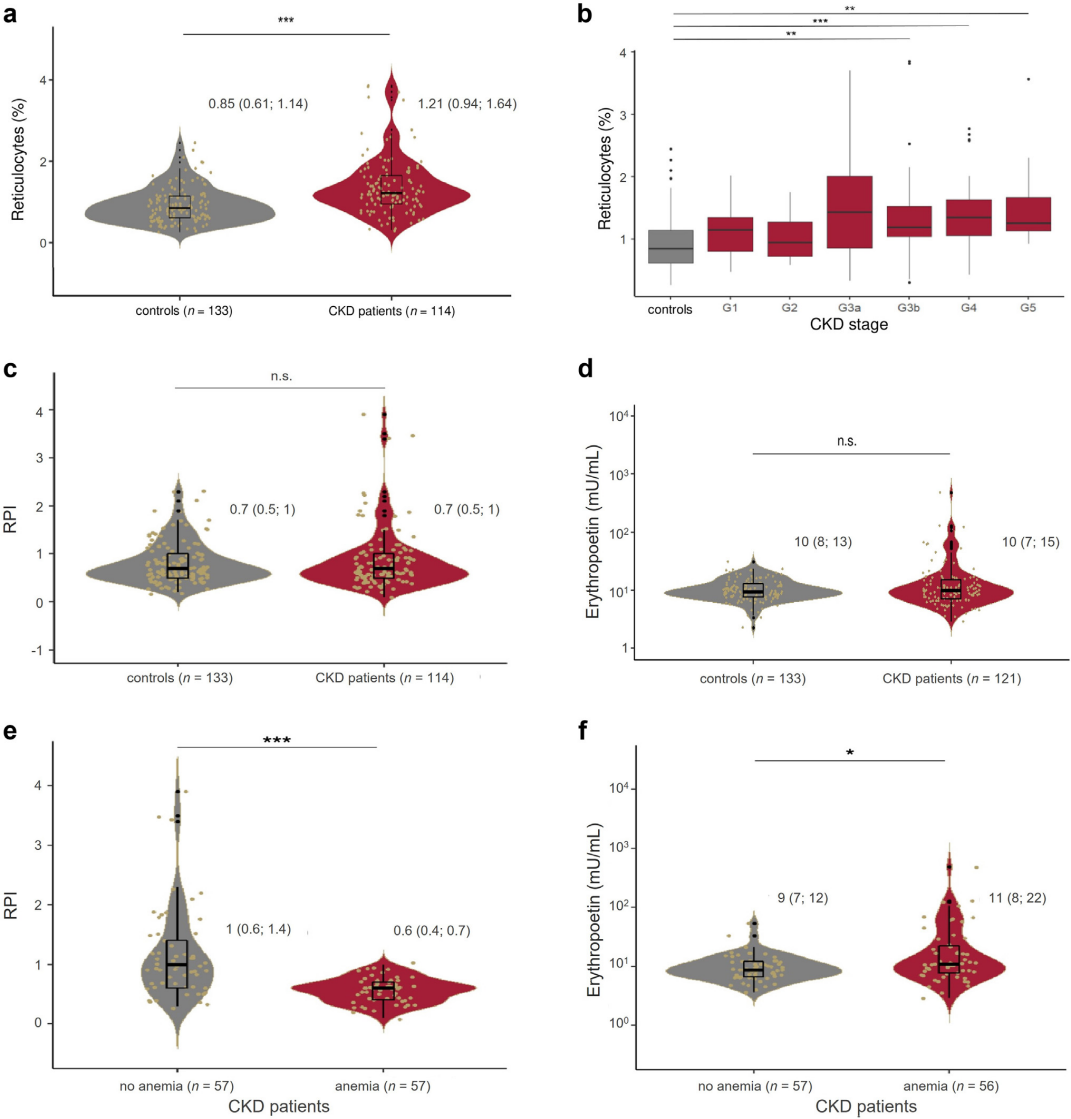
Erythropoiesis and EPO secretion is inadequate for the degree of anemia in patients with CKD. (a) Violin plots of the reticulocyte count in the control and CKD group. (b) Reticulocytes according to CKD stages. (c) Violin plots of RPI in the control and CKD group. RPI is 1 in the absence of anemia, whereas an RPI > 3 indicates a normal response and a RPI < 2 is an inadequate response to anemia. (d) Plasma EPO concentration in the control and CKD group (note the logarithmic scale of the y-axis). (e and f) RPI and plasma EPO concentration in patients with CKD according to the presence of anemia (note the logarithmic scale of the y-axis). CKD, chronic kidney disease; EPO, erythropoietin; RPI, reticulocyte production index.

Physiologically, anemia is expected to stimulate counterregulatory increase in erythropoiesis reflected by increased renal EPO secretion and reticulocyte count. Determination of the reticulocyte count by FACS demonstrated a slightly but significantly increased count in patients with CKD when compared with the healthy group (Figure 2a). This increase was observed to be higher in higher GFR stages of CKD (Figure 2b). When calculating the reticulocyte production index, which quantifies the adequacy of erythropoiesis in relation to anemia, patients with CKD had an insufficiently increased reticulocyte production index (Figure 2c). These observations matched the plasma EPO concentration, which was not increased in the CKD group as compared with the healthy group (Figure 2d). However, EPO concentration exceeded 30 mU/ml in 1 healthy donor and in 14 patients with CKD, out of which 4 patients were receiving erythropoiesis stimulating agent (ESA) therapy. When comparing patients with CKD with or without anemia, reticulocyte production index was significantly lower whereas plasma EPO concentrations were slightly increased in those with anemia (Figure 2e and f).

### Quantification of RBC Death as Represented by the Annexin V-Binding

**Figure 3:**
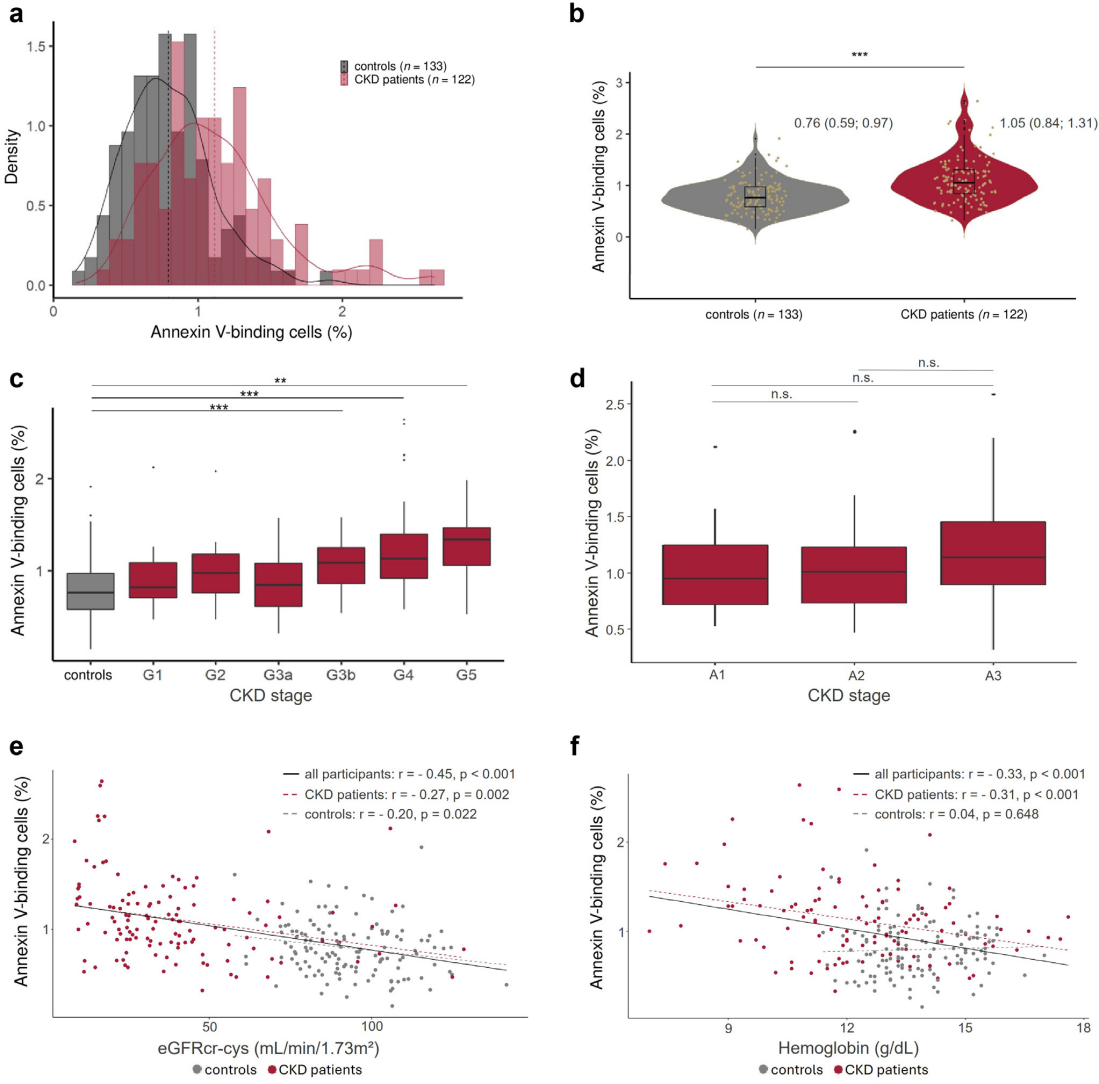
RBC death represented by annexin V-binding increases with progressive CKD. (a and b) Density function and violin plot of the percentage of annexin V-binding RBCs in control and patients with CKD. (c and d) Percentage of annexin V-binding RBCs as per CKD GFR and albuminuria stages. (e and f) Correlation of the percentage of annexin V-binding RBCs with eGFR and hemoglobin concentration in all participants, including both the control and CKD groups, as well as within the control and CKD groups separately. CKD, chronic kidney disease; eGFR, estimated glomerular filtration rate; RBC, red blood cell.

The rate of RBC death was significantly higher in patients with CKD when compared with control individuals (Figure 3a and 3b). The increase in RBC death rate was associated with higher GFR stage of CKD and the difference became significantly different between control individuals and patients with CKD of the stages G3b and higher (Figure 3c). RBC death rate tended to be increased with higher albuminuria stages in patients with CKD, although the increase did not reach statistical significance (Figure 3d). No significant difference in the median RBC death rate was observed between female and male healthy individuals (0.7% [0.5%–0.9%] vs. 0.8% [0.7%–1.0%], *P* = 0.092). Similarly, the RBC death rate in female patients with CKD (1.0% [0.8%–1.3%]) did not notably differ from the RBC death rate of male patients with CKD (1.1% [0.9%–1.4%], *P* = 0.373). We observed a significant negative correlation between the percentage of annexin V-binding RBCs with eGFR (*r* = −0.45, *P* < 0.001, Figure 3e) and Hb concentration (*r* = −0.33, *P* < 0.001, Figure 3f) across the entire cohort. These correlations were driven by the correlation in the CKD subgroup (RBC death rate and eGFR: *r* = −0.27, *P* = 0.002; RBC death rate and Hb: *r* = −0.31, *P* < 0.001). In the control group, there was also a negative correlation between eGFR and RBC death rate (*r*= −0.20, *P* = 0.02).

Given previous research conducted among patients on hemodialysis and peritoneal dialysis^25^^,^^26^ demonstrating the presence of components within plasma that might foster RBC death, we undertook the incubation of healthy RBCs in plasma of patients with CKD, and in plasma derived from healthy control subjects. Subsequent analysis of the incubated RBCs after 24 hours revealed a noteworthy increase in RBC death rates for both RBCs incubated with patient plasma (5.7% [4.0%–8.7%]) and those in control subjects’ plasma (5.7% [4.1%–7.8%], *P* = 0.478), with the increase in RBC death in the control group being normal and comparable to findings in a previous study by our group.^26^ A subanalysis of incubated plasma stratified by GFR stage of CKD provided the following results: (5.7% [3.7%–6.1%] in stage G1, 5.5% [3.8%–7.5%] in stage G2, 5.4% [4.8%–8.7%] in stage G3a, 6.9% [4.4%–10.9%] in stage G3b, 5.1% [3.8%–8.8%] in stage G4, and 7.7% [4.1%–9.6%] in stage G5. Although no consistent trend was observed across stages G1 to G4, the highest percentage of RBC death was recorded in healthy erythrocytes incubated with plasma from stage G5. However, no significant differences were observed between the groups. In addition, eGFR and RBC death in plasma were not significantly correlated in the overall cohort (*r* = −0.09, *P* = 0.16) or in the patient subgroup (*r* = −0.08, *P* = 0.376), although a similar trend was observed in both.

### Cellular Markers of RBC Death

The generation of ROS, ceramide, and intracellular calcium have been established as significant factors involved in RBC death.^32^ Therefore, we proceeded to explore whether these markers might be induced in RBCs of patients with CKD compared with healthy controls. The median of the geometric mean fluorescence intensity of the intracellular calcium concentration and ROS did not show a significant difference to the normalized value of the control group (15.2 [13.2–17.6] vs. 14.9 [14.8–15], *P* = 0.461). However, we did observe an elevated geometric mean fluorescence intensity of ceramide in the CKD group compared with the control group (15.5 [14.0–16.6] vs. 15.0 [14.9–15.1], *P* = 0.002).

### Factors Associated With RBC Death

In the overall cohort, a univariate positive correlation emerged between annexin V-binding and plasma creatinine and cystatin C concentration, whereas there was a negative correlation with hematocrit and erythrocyte count (Table 3).

**Table 3:** Correlations of RBC death represented by the percentage of annexin V-binding with laboratory parameters in the overall cohort and in patients with CKD and upper and lower 95% confidence intervals

Parameter	Univariate		Multivariable
	All participants *(n* = 255)	Only CKD (*n* = 122)	All participants with full dataset (*n* = 246)
Age, years (CI)	0.14^a^ (0.02–0.26)	−0.01, n.s. (−0.18 to 0.17)	n.s.
Sex (0 = male, 1 = female) (CI)	0.1, n.s. (−0.02 to 0.22)	0.1, n.s. (−0.08 to 0.27)	n.s.
Plasma creatinine, mg/dl (CI)	0.44^b^ (0.33–0.53)	0.34^b^ (0.17–0.48)	n.s.
Plasma cystatin C, mg/l (CI)	0.48^b^ (0.38–0.57)	0.36^b^ (0.2–0.51)	n.s.
eGFR cr-cys, ml/min per 1.73m^2 (CI)^	−0.45^b^ (−0.55 to −0.35)	−0.27^c^ (−0.43 to −0.1)	b
Hemoglobin, g/dl (CI)	−0.33^b^ (−0.44 to -0.21)	−0.31^b^ (−0.46 to −0.13)	n.s.
Hematocrit, % (CI)	−0.36^b^ (−0.46 to −0.24)	−0.31^b^ (−0.47 to −0.14)	n.s.
Erythrocytes, 10^6^/μl (CI)	−0.34^b^ (−0.44 to −0.22)	−0.34^b^ (−0.49 to −0.17)	n.s.
MCV, fL (CI)	0.03, n.s. (−0.1 to 0.15)	0.18, n.s. (−0.01 to 0.35)	n.s.
MCH, pg (CI)	0.09, n.s. (−0.06 to 0.23)	0.13,n.s. (−0.06 to 0.3)	n.s.
Plasma CRP, mg/dl (CI)	0.08, n.s. (−0.05 to 0.2)	0.003,n.s. (−0.18 to 0.18)	n.s.
Plasma iron, μg/dl (CI)	−0.2^c^ (−0.31 to −0.08)	−0.03, n.s. (−0.21 to 0.14)	n.s.
Plasma ferritin, μg/dl (CI)	0.19^c^ (0.06–0.3)	0.07, n.s. (−0.11 to 0.24)	n.s.
Plasma transferrin, mg/dl (CI)	−0.25^b^ (−0.37 to −0.14)	−0.11, n.s. (−0.28 to 0.07)	n.s.
TSAT, % (CI)	−0.09, n.s. (−0.21 to 0.03)	0.004, n.s. (−0.17 to 0.18)	n.s.
Plasma EPO, mU/ml (CI)	0.05, n.s. (−0.07 to 0.17)	0.003, n.s. (−0.18 to 0.18)	n.s.
Plasma total protein, g/dl (CI)	n.d.	−0.15,n.s. (−0.36 to 0.06)	n.s.
Urinary protein, mg/g creatinine (CI)	n.d.	0.01, n.s. (−0.17 to 0.19)	n.s.
Urinary albumin, mg/g creatinine (CI)	n.d.	0.12, n.s. (−0.07 to 0.29)	n.s.

CI, 95% confidence interval; CKD, chronic kidney disease; CRP, C-reactive protein; eGFR, estimated glomerular filtration rate; EPO, erythropoietin; MCH mean corpuscular hemoglobin; MCV mean corpuscular volume; n.d., no data; n.s. not significant; TSAT, transferrin saturation.

*P* > 0.05 has been considered as not significant (n.s.).

a *P* < 0.05.

b *P* < 0.001.

c *P* < 0.01.

Across the entire cohort, plasma ferritin was positively correlated with annexin V-binding (*r* = 0.19, *P* = 0.003, Supplementary Figure 2E), whereas there was a negative correlation of plasma iron (*r* = −0.2, *P* = 0.002, Supplementary Figure 2F) and transferrin concentration with annexin V-binding within the overall cohort (*r* = −0.25, *P* < 0.001, Table 3).

**Figure 4:**
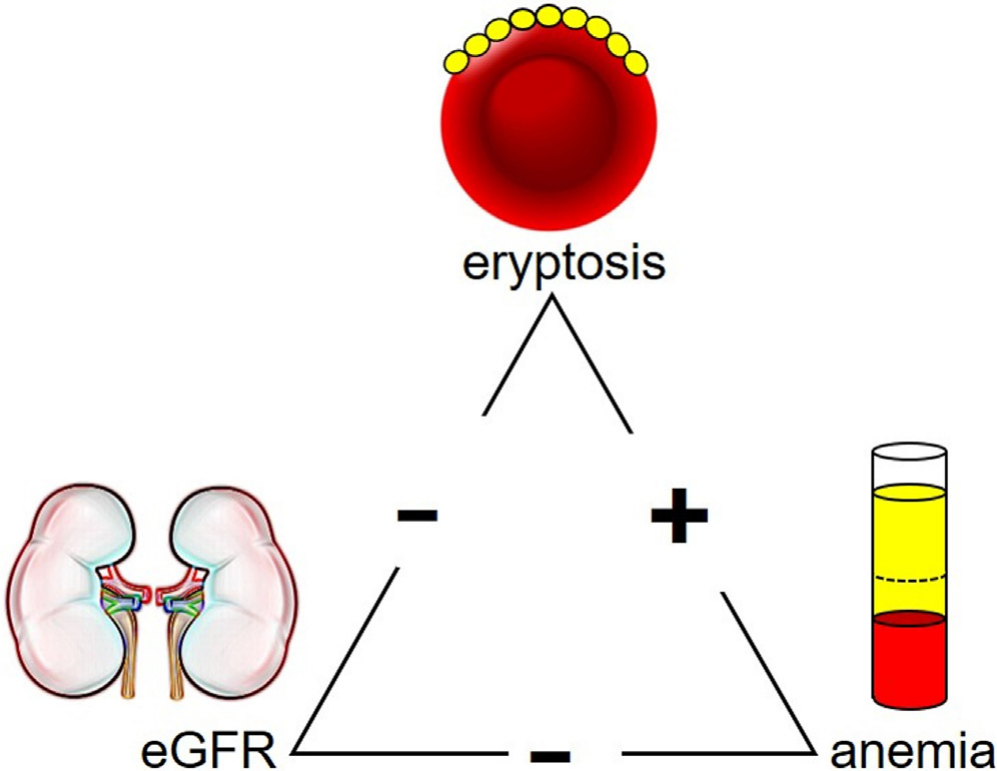
Schematic illustration of the interplay between eryptosis, eGFR, and anemia. The schematic diagram elucidates the intricate relationship among eryptosis, eGFR, and anemia. The eryptosis rate is negatively correlated with eGFR and positively with anemia. In addition, eGFR is negatively correlated with anemia. This suggests that eryptosis is associated with progressive CKD and anemia. CKD, chronic kidney disease; eGFR, estimated glomerular filtration rate.

To identify independent predictors of RBC death in patients with CKD, multivariable linear regression was employed involving the complete cohort, including 246 individuals with the full dataset (113 patients with CKD and 133 control subjects). To exclude confounding effects, none of the medication groups or comorbidities were included in the model. Among the parameters integrated into the stepwise analysis in the entire cohort, only eGFR entered the final model and was identified as the sole predictor of RBC death rate, represented by the percentage of annexin V-binding. The model exhibited moderate goodness of fit, with an *r*^*2*^ = 0.22 (adjusted *r*^*2*^ = 0.21).^33^ When only patients with CKD were analyzed, similar results were obtained as in the entire cohort: among the parameters integrated into the stepwise analysis, only eGFR entered the final model and was identified as the only significant predictor of RBC death rate (*r*^*2*^= 0.1; adjusted *r*^*2*^= 0.09). It is noteworthy that the slope of the association of eGFR with annexin V-binding was negative, indicating higher RBC death with progressive eGFR decline (Figure 4). In contrast, RBC death and anemia were positively correlated, demonstrating a strong association between anemia and enhanced RBC death. In addition, eGFR and anemia correlated negatively with each other (Figure 4).

Because inflammation is recognized as a principal factor influencing RBC death,^21^ we conducted correlation analyses to explore its impact. Our findings revealed no association between plasma C-reactive protein concentration and RBC death in either the entire cohort or the patient subgroup. In addition, we investigated the correlation between C-reactive protein and eGFR in the patient group and observed no significant relationship. Consequently, we inferred that the presence of inflammation did not influence the observed results.

## Discussion

Our study reveals compelling evidence that NDD-CKD is associated with enhanced RBC death represented by annexin V-binding of freshly drawn RBCs. Specifically, the RBC death rate among patients with CKD was nearly 1.4 times higher than in healthy controls. Our results demonstrating a gradual increase in RBC death rate with progressive CKD fit well to previous results obtained in other studies. Bonan *et al.* for example explored the mechanisms of RBC death in CKD stages 3 to 4, but their study included only 6 patients.^27^ Bonomini *et al.* analyzed RBC death in 30 patients with CKD not categorized according to the current CKD stages.^28^ Recently, Gok *et al.* observed increased RBC death in a composite group of 59 patients with CKD stages 3 to 5.^29^ Although the aforementioned studies similarly examined RBC death in NDD-CKD, they either did not investigate all CKD stages or included only a small number of patients. To the best of our knowledge, our study stands as the first to identify eGFR as the independent predictor for stimulation of RBC death, with lower eGFR values associated with higher RBC death. These results are in line with previous results obtained from patients on dialysis with minimal or even absent eGFR, demonstrating a RBC death rate twice as high in comparison to healthy individuals.^25^^,^^26^ Given the swift elimination of PS-positive RBCs from the circulation, it can be assumed that a doubling of the RBC death rate halves the RBC lifespan, which is approximately 126 days in healthy individuals.^25^^,^^26^^,^^34^ Li *et al.* determined the RBC lifespan in patients with CKD to be gradually reduced from approximately 122 days in CKD stage 1 to approximately 60 days in CKD stage 5.^35^ Our results demonstrating an intermediate increase in RBC death rate with progressive CKD, thus, fit well to these results. Physiologically, a reduced RBC lifespan should stimulate erythropoiesis by increased renal EPO secretion. However, in CKD, there is an inadequately low reticulocyte count and an insufficient renal EPO secretion (Figure 2).^15^ Therefore, in CKD, impaired erythropoiesis synergizes with reduced RBC lifespan and inevitably leads to anemia.

Previous studies in patients on dialysis have underscored elevated intracellular calcium concentrations, oxidative stress, and enhanced ceramide formation as well-established triggers of RBC death.^25^^,^^26^^,^^36^^,^^37^ Intriguingly, we noted an increased ceramide concentration within our patient cohort, and patients displayed a tendency toward higher ROS levels as compared with the respective controls. However, the latter discrepancy lacked statistical significance. Moreover, a significant negative correlation emerged between ROS and eGFR, mirroring the heightened oxidative stress observed in the context of declining kidney function.^38^ Conversely, although no measurable increase in intracellular calcium concentration was detected in patients with CKD, there was a significant negative correlation between intracellular calcium concentration and patients’ Hb concentrations. Despite the lack of significant difference in intracellular calcium levels in our cohort, the negative correlation with Hb suggests a potential link between intracellular calcium dysregulation and anemia severity. In contrast, Abed *et al.* studied patients with dialysis-dependent CKD and reported elevated intracellular calcium concentrations. Notably, the patients in the study of Abed *et al.* had Hb concentrations more than 2 g/dl lower than those in our cohort, which may account for the observed increase in intracellular calcium in their study. This suggests that the severity of anemia, particularly in dialysis-dependent patients, might contribute to the differences in intracellular calcium levels.^26^

Previous studies in patients on dialysis also demonstrated increased PS exposure in healthy RBCs after a 24-hour incubation in plasma from patients on dialysis but not after incubation in plasma from healthy individuals.^25^^,^^26^ These findings led to the speculation that uremic plasma components could potentially act as triggers for RBC death, especially the uremic toxins including urea, indoxyl sulfate, and p-cresyl sulfate, which have been found to trigger RBC death.^32^ However, in our study, when assessing RBCs incubated in plasma for 24 hours, no differences were noted between RBCs incubated in patient plasma versus healthy plasma. This could be attributed to relatively lower uremic toxin concentrations in patient plasma, because of better residual renal function. Virzi *et al.* observed that patients with residual diuresis exhibited lower RBC death rates compared with those without diuresis.^39^ This finding was attributed to the lower concentration of uremic toxins associated with better kidney function.^39^ Abed *et al.* also speculated that plasma components influencing RBC death might be removed or inactivated during dialysis, because they witnessed a decline in the effect of uremic plasma on control RBCs post hemodialysis.^26^ Overall, the effect of such plasma components might not be as pronounced in the current CKD cohort with a median eGFR of 33 ml/min per 1.73 m^2^. Other factors associated with CKD, which may reduce RBC survival include oxidative stress,^23^ hyperphosphatemia,^40^ and impaired nitric oxide bioavailability.^41^^,^^42^

As outlined by Kidney Disease Improving Global Outcomes, iron deficiency anemia ranks among the frequent and reversible causes of chronic or progressive anemia in CKD.^3^ In line with this, 40 (33%) of the patients with CKD in our study exhibited iron deficiency, with over half of them (*n* = 24, 21%) experiencing iron deficiency anemia. Notably, both iron and transferrin levels declined in a CKD-stage dependent manner, correlating positively with eGFR and Hb levels. Within the entire cohort, negative correlations were found between iron and transferrin concentrations and the RBC death rate. This agrees with a study by Kempe *et al.*, who demonstrated increased RBC death rates in iron-deficient mice.^43^ However, iron status was not found to be a significant predictor of RBC death when analyzed using multiple linear regression, indicating that other factors may have a stronger influence on RBC survival in patients with CKD. This suggests that the relationship between iron status and RBC death in the context of CKD is complex and may involve additional contributing factors. Further research is necessary to better understand how iron metabolism interacts with mechanisms of eryptosis and ferroptosis in CKD. Eryptosis and ferroptosis are distinct processes involving cell death, with eryptosis referring to the death of RBCs and ferroptosis being a form of regulated cell death dependent on iron levels.^44^ Investigating the interplay between these processes could provide insights into novel therapeutic targets for managing anemia in patients with CKD.

Currently, ESAs are a mainstay of the treatment of renal anemia. However, a full correction cannot be achieved with reasonable ESA doses. This might be accounted for by the persistence of the increased RBC death rate in CKD. A previous study in patients on dialysis found a positive correlation of ESA dose with annexin V-binding, suggesting higher ESA requirements in patients with strongly stimulated RBC death.^25^ However, excessively high ESA dosages carry an increased risk of thrombosis^45^ and microcirculation disturbances, possibly linked to increased adhesion of PS-exposing RBCs to the vascular wall and platelets, further hampering blood flow.^45^ Moreover, augmented PS exposure correlates with heightened blood coagulation.^46^ Therefore, a magnified RBC death rate might exacerbate the already elevated cardiovascular risk in patients with CKD,^33^ contributing partially to the adverse events witnessed during high-dose ESA therapy.^47^ Consequently, an intervention aiming at inhibition of RBC death alongside stimulation of erythropoiesis would be promising. However, more research is required to elucidate the exact mediators of RBC death in progressive CKD, which might be targeted therapeutically.

A few limitations are acknowledged in our study. First, being a cross-sectional study, it provides a snapshot of the relationship between RBC death, anemia, and CKD but cannot establish causality. Longitudinal studies are needed to elucidate the temporal relationship between these factors. Second, our study focused on patients with NDD-CKD, and therefore, the findings may not be directly applicable to patients with acute kidney injury. Third, the sample size, although including 122 patients, might not capture all possible confounders influencing RBC death in CKD. Fourth, though we assessed various markers associated with RBC death, other potential mediators or pathways contributing to eryptosis in CKD remain unexplored. Fifth, the incubation experiments with healthy RBCs in patient plasma might not fully represent the *in vivo* environment in patients with CKD. Finally, our study did not investigate the impact of potential treatments or interventions targeting RBC death, which warrants further investigation to explore therapeutic opportunities.

In conclusion, this study highlights the stimulation of RBC death in NDD-CKD in parallel to progressive GFR decline, which is possibly contributing to the development of renal anemia.

## Disclosure

All the authors declared no competing interests.

## Supplementary Material

Supplementary File (PDF) **Figure S1.** Representative histogram from FACS analysis for annexin V-binding of RBCs. **Figure S2.** Iron deficiency and iron deficiency anemia according to the CKD stage.

## Footnotes

Supplementary File (PDF)
**Figure S1.** Representative histogram from FACS analysis for annexin V-binding of RBCs. **Figure S2.** Iron deficiency and iron deficiency anemia according to the CKD stage.
