# Supplementary material for: GFR is a Key Determinant of Red Blood Cell Survival in Anemia Associated With Progressive CKD
Source: Kidney Int Rep. 2024 Dec 21;10(3):730–42. doi: 10.1016/j.ekir.2024.12.023 (PMC11993223; doi:10.1016/j.ekir.2024.12.023)

Supplementary material

Supplementary Figures

Supplementary Figure 1: Representative histogram from FACS analysis for Annexin-V binding of RBCs

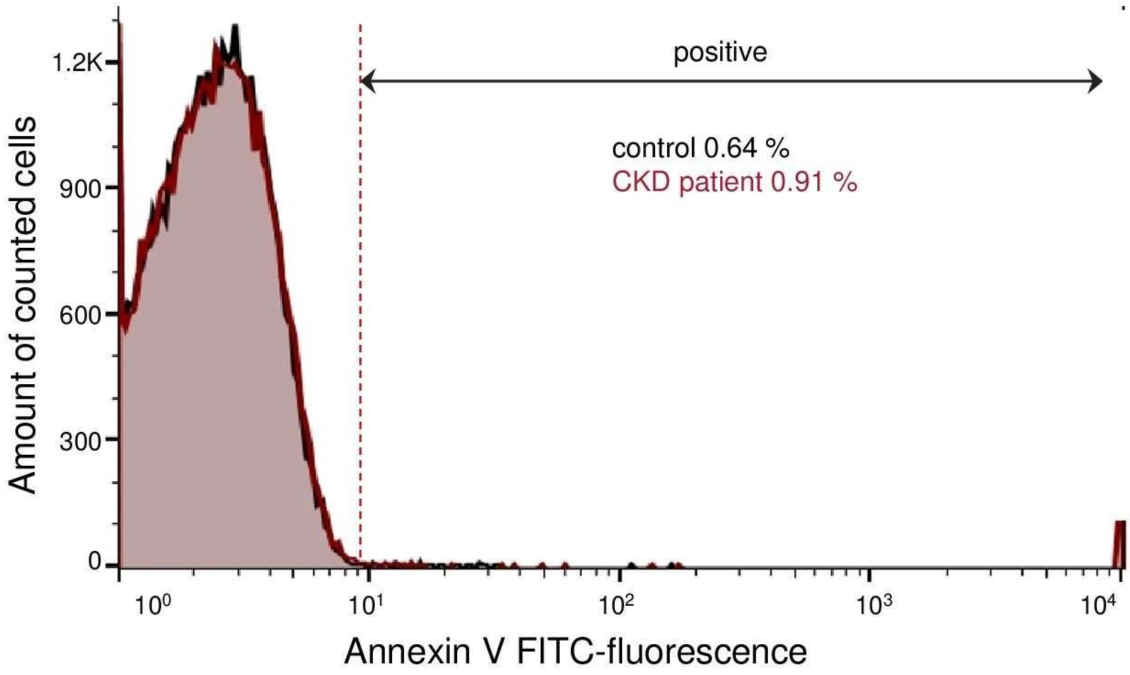

Supplementary Figure 2: Iron deficiency and iron deficiency anemia according to the CKD stage

(A) Absolute number of patients with/ without iron deficiency (percentage of patients in each CKD stage), a total of 122 CKD patients, iron deficiency defined as transferrin saturation ≤ 20% and ferritin ≤ 10 µg/dL [3].

(B) Absolute number of patients with/ without iron deficiency anemia (percentage of patients in each CKD stage), a total of 122 CKD patients, out of which n = 8 patients had unknown hemoglobin (Hb) values. Anemia defined based on sex specific Hb values, iron deficiency defined as stated above.

(C) Absolute number of patients with/ without iron deficiency (percentage of patients in each albuminuria stage), a total of 122 CKD patients, iron deficiency defined as transferrin saturation ≤ 20% and ferritin ≤ 10 µg/dL, n = 5 patients had unknown albuminuria values.

(D) Absolute number of patients with/ without iron deficiency anemia (percentage of patients in each albuminuria stage), a total of 122 CKD patients, out of which n = 8 patients had unknown hemoglobin (Hb) values and n = 5 patients unknown albuminuria values. Anemia defined based on sex specific Hb values, iron deficiency defined as stated above.

(E) Positive correlation between plasma ferritin and annexin V binding in the overall cohort

(F) Negative correlation between plasma iron and annexin V binding in the overall cohort

Supplementary Figure 2

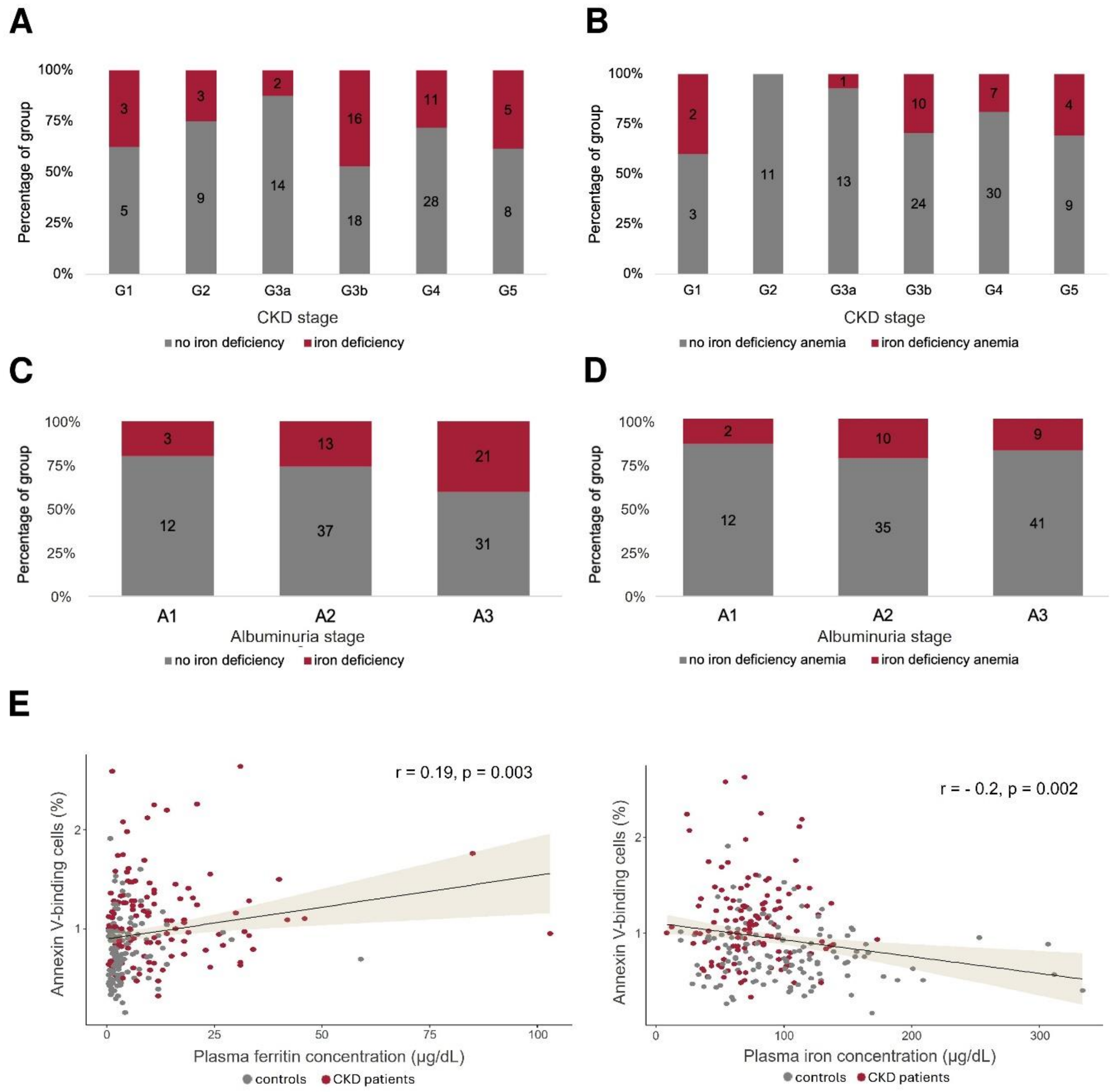

Supplement: Supplementary File (PDF) — Figure S1. Representative histogram from FACS analysis for annexin V-binding of RBCs. Figure S2. Iron deficiency and iron deficiency anemia according to the CKD stage. [file mmc1.pdf]
